# Hyperoxaemia in acute trauma is common and associated with a longer hospital stay: a multicentre retrospective cohort study

**DOI:** 10.1186/s13049-024-01247-5

**Published:** 2024-08-21

**Authors:** Manuela Iten, Urs Pietsch, Juergen Knapp, Dominik Andreas Jakob, Gert Krummrey, Christian Maschmann, Jacob Steinmetz, Tobias Arleth, Martin Mueller, Wolf Hautz

**Affiliations:** 1 Department of Intensive Care Medicine, Inselspital, University Hospital Bern, Bern, Switzerland; 2 Division of Perioperative Intensive Care Medicine, Cantonal Hospital St.Gallen, St. Gallen, Switzerland; 3 Swiss Air Rescue, Rega, Zurich, Switzerland; 4 Department of Emergency Medicine, Inselspital, University Hospital Bern, Bern, Switzerland; 5 Department of Anaesthesiology and Pain Medicine, Inselspital, University Hospital Bern, Bern, Switzerland; 6 Institute for Medical Informatics I4MI, Bern University of Applied Sciences, Biel/Bienne, Switzerland; 7 Department of Emergency Medicine, Cantonal Hospital of St. Gallen, St. Gallen, Switzerland; 8 Department of Anaesthesia, Centre of Head and Orthopaedics, University of Copenhagen, Rigshospitalet, Denmark; 9 Danish Air Ambulance, Aarhus, Denmark

**Keywords:** Trauma, Oxygen, Hyperoxaemia, Normoxaemia, Hypoxaemia

## Abstract

**Background:**

Trauma poses a significant global health challenge. Despite advancements in the management of severely injured patients, (poly)trauma continues to be a primary contributor to morbidity and mortality worldwide. In the context of trauma resuscitation, supplemental oxygen is commonly administered generously as suggested by guidelines. Yet, it remains uncertain whether the trauma population might derive advantages from a more conservative approach to supplemental oxygen.

**Methods:**

In this retrospective cohort study from two Swiss trauma centers, severely injured adult (> 16 years) trauma patients with an Injury Severity Score (ISS) ≥ 16 were divided into four groups according to the first blood gas analysis taken: hypoxaemia (PaO_2_ < 10.7 kPa/80 mmHg), normoxaemia (PaO_2_ 10.7–16.0 kPa/80–120 mmHg), which served as reference, moderate hyperoxaemia (PaO_2_ > 16.0–40 kPa/120–300 mmHg) and severe hyperoxaemia (PaO_2_ > 40 kPa/300 mmHg). The primary outcome was 28-day mortality. Length of hospital stay (LOS) and length of intensive care unit stay (LOS-ICU) were analyzed as secondary outcomes.

**Results:**

Of 1,189 trauma patients, 41.3% had hyperoxaemia (18.8% with severe hyperoxaemia) and 19.3% had hypoxaemia. No difference was found for 28-day mortality (hypoxaemia: 15.7%, normoxaemia: 14.1%, hyperoxaemia: 13.8%, severe hyperoxaemia: 16.0%, *p* = 0.846). Patients with severe hyperoxaemia had a significant prolonged LOS (median 12.5 [IQR 7–18.5] days vs. 10 [7–17], *p* = 0.040) and extended LOS-ICU (3.8 [1.8–9] vs. 2 [1–5] days, *p* = 0.149) compared to normoxaemic patients. In multivariable analysis, oxygen group was not associated with the primary outcome 28-day mortality or LOS-ICU. Severe hyperoxaemia patients had a tendency towards longer hospital stay (adjusted coefficient 2.23 days [95% CI: − 0.32; 4.79], *p* = 0.087).

**Conclusion:**

Hyperoxaemia was not associated with an increased 28-day mortality when compared to normoxaemia. However, both moderate and severe hyperoxaemia is frequently observed in trauma patients, and the presence of severe hyperoxaemia showed a tendency with extended hospital stay compared to normoxaemia patients. Robust randomized controlled trials are imperative to thoroughly evaluate the potential correlation between hyperoxaemia and outcomes in trauma patients .

*Trial Registration* Retrospectively registered.

**Supplementary Information:**

The online version contains supplementary material available at 10.1186/s13049-024-01247-5.

## Background

Trauma represents a major global health issue and has been identified as a key concern for health care by the World Health Organization (WHO) [1]. Despite advances in care of severely injured patients, (poly-)trauma remains a leading cause of morbidity and mortality worldwide [2–4].

In trauma resuscitation, supplemental oxygen is often administered to treat and prevent hypoxaemia as recommended by the Advanced Trauma Life Support (ATLS) and the Pre-hospital Trauma Life Support (PHTLS) manual [5, 6]. Oxygen is cheap and widely available in the prehospital as well as in the clinical setting. In general, it is believed that oxygen therapy – at least if administered over a short period of time – is of little harm. This leads to a liberal use of supplemental oxygen in trauma patients and results in hyperoxaemia as a common finding in trauma patients [7]. Moreover, it is frequently administered in the prehospital setting even without an indication [8].

A recent systematic review found extremely sparse evidence for or against the use of supplemental oxygen in the trauma population [9]. Knowledge on the use of supplemental oxygen mainly originates from other critical patient populations and not specifically from trauma patients. However, emerging evidence indicates that even modest hyperoxaemia in general might be harmful. In a systematic review and meta-analysis, Chu et al. compared a liberal versus a restrictive oxygen strategy for a broad mix of acutely ill medical and surgical patients and found an association between liberal oxygen administration and increased mortality [10]. In addition to mortality, hyperoxaemia has been associated with major pulmonary complications in the intensive care unit (ICU) as well as in surgical patients [11, 12]. In contrast, a recent study that randomized ICU patients to either low or high oxygenation levels revealed no discernible difference in mortality rates [13].

For trauma patients, a study on 68 traumatic brain injury patients showed that the degree of disability was significantly reduced at six months in the group receiving liberal compared to restrictive oxygen [14]. In contrast, Douin et al. found an association between higher fraction of inspired oxygen (FiO2) administration during periods of hyperoxaemia (saturation 100%) and greater risk of mortality among critically injured patients [15]. It remains uncertain whether the trauma population could benefit from a more restrictive supplemental oxygen approach than recommended by current international guidelines.

In our retrospective cohort study, we aimed to assess mid-term mortality (28-day) of stratified oxygen groups of hypoxaemic, normoxaemic, hyperoxaemic, and severe hyperoxaemic trauma patients. We hypothesized that in the trauma population, hypo- and hyperoxaemia are common and associated with increased mortality compared to normoxaemia.

## Methods

This retrospective cohort study was performed in two level one trauma centers in Switzerland; Inselspital, University Hospital of Bern and Hospital of Canton St. Gallen (KSSG). The study protocol was approved by the local cantonal ethics committee of the Canton of St. Gallen (approval EKOS 23/178, project number 2023-01801). This report follows the applicable STROBE guidelines [16].

Anonymized patient data from the electronic health records in both hospitals were screened for all patients that presented to Inselspital Bern or KSSG for acute trauma care between 01.01.2017 and 31.12.2022. Screening included coding of free text data through standard trauma scoring systems such as Injury Severity Index (ISS) [17, 18] and abbreviated injury scale (AIS) [19]. Inclusion criteria were primary admission with trauma and ISS ≥ 16, age ≥ 16 years old, and arterial blood gas analysis (aBGA) performed within 3 h after admission. Exclusion criteria was missing PaO_2_ (arterial partial pressure of oxygen) value in the initial aBGA and missing information on prehospital airway status (intubated or not). Severe trauma to a specific body region was defined as having a maximum AIS of three or higher for that region.. Demographic data, pre-hospital and clinical data as well as details on injury were collected.

Based on the first available result of PaO_2_ within the first 3 h after admission, patients were divided into four groups [20]:

- Group 1 hypoxaemia: all patients with PaO_2_ < 10.7 kPa (80 mmHg)
- Group 2 normoxaemia: all patients with PaO_2_ between 10.7 and 16.0 kPa (80–120 mmHg)
- Group 3 mild hyperoxaemia all patients with PaO_2_ above 16.0 to 40 kPa (120–300 mmHg)
- Group 4 severe hyperoxaemia: all patients with PaO_2_ above 40 kPa (300 mmHg)

Normoxaemic patients (group 2) served as reference group. Demographic data, pre-hospital and clinical data and injury scores were calculated and compared for all four groups.

### Statistics

As primary outcome, 28-day mortality was assessed and compared across groups, with group 2 (normoxaemia) as reference. For secondary outcomes, length of ICU stay (LOS-ICU) and length of hospital stay (LOS) were calculated and compared between groups. A predefined set of complications (stroke, myocardial infarction, pulmonary embolism, deep vein thrombosis, pressure ulcer, renal insufficiency, abdominal distension, wound infection, pneumonia, urinary tract infection, sepsis, compartment syndrome (extremity), abdominal compartment syndrome, acute lung injury (ALI) / acute respiratory distress syndrome (ARDS), cardiac arrest, multiorgan failure) were reported.

Data were analyzed with STATA 18.1 (StataCorp, College Station, TX, USA). For primary and secondary endpoints, odds ratios (OR) were calculated in addition to 95% confidence intervals for all point estimates. Depending on normality testing (Shapiro Wilk) median (interquartile range (IQR)) respectively mean (standard deviation (SD)) are shown for continuous variables, p-values obtained by Kruskal–Wallis test (for more than two groups) or Wilcoxon rank sum test (for two groups). Categorical variables are shown with number (%) in each category, p-values obtained by Chi-squared test. No p-value adjustment was performed.

Baseline data was used to derive a multivariable logistic (linear) regression model for the association between the four oxygen groups and the primary (secondary) endpoint as follows:

If binary variables were based on present continuous variables (such as hypotension on systolic blood pressure), the continuous parameter was used only to derive to the final model to avoid collinearity. Furthermore, oxygen saturation and GCS were not included in the multivariable model based on collinearity testing with oxygen group respectively AIS head (Variance Inflation Factor of > 2.5). Missing values were imputed and a stepwise backward regression analysis with a p-value of 0.05 was used to get to the final model. Oxygen group was forced in the final model. Logistic regression associations were presented as adjusted OR (aOR), linear regression associations with the coefficient accompanied with a 95% confidence interval (CI). Last, to explore non-linear associations of PaO_2_ and 28-day mortality, a fractional polynomial regression was used in one dimension with different powers (− 2, − 1, − 0.5, 0 = ln, 0.5 1, 2, 3) adjusted for the same co-variables as the final model. The proposed model was visualized with an adjusted predicted probability plot.

With a total of an expected 1200 trauma patients (around 200 patients per year), with 300 patients in each of the four oxygen groups, we would have been able to detect a clinical meaningful difference of 7% in 28-day mortality between normoxaemia (14%) and severe hyperoxaemia (21%).

## Results

### Study population

**Fig. 1:**
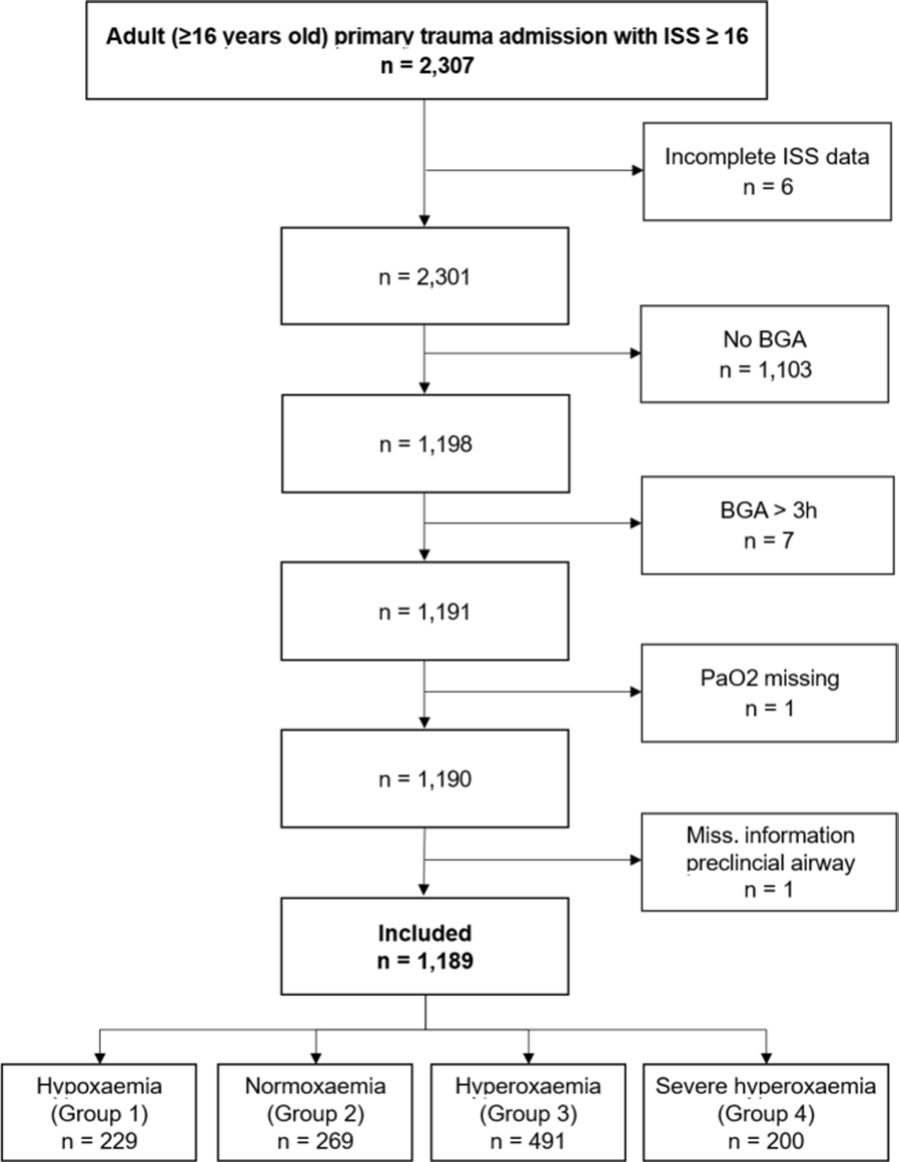
Flow Diagram of included patients. BGA, Blood Gas Analysis; ISS, Injury Severity Index; Miss., missing

From January 2017 to December 2022, a total of 1,189 adult patients with an ISS of 16 or higher, who were primarily admitted to the two emergency departments, were included in the primary analysis (Fig. 1).

The baseline characteristics according to the oxygen group are shown in Table 1. The median age was 57 [IQR 40–73] years and 27.8% were female in this cohort. Patients in the severe hyperoxaemia group were significantly younger (51.5 [30.5; 68.5] years), and more frequently female (37.0%), overall p-value = 0.001. They presented with a higher median AIS head score (3, [0–5], *p* = 0.019) and lower GCS (5.5, [3–14], overall *p* ≤ 0.01). Baseline data for blood pressure and respiratory rate showed statistically significant, clinically negligible differences between the groups. More patients in both the hyperoxaemia (30.8%) and the severe hyperoxaemia (50.0%) group were intubated prehospital compared to normoaemic (20.8%) and hypoxaemic (17.9%) patients. Severe thoracic trauma (AIS thorax > 2) was more prevalent in the normoxaemia and hypoxaemia group (52.8% and 59.4%, respectively) compared to the moderate and severe hyperoxaemia groups (46.4% and 40.0%, supplemental Table 1). The total population is shown in supplement Table 2.

**Table 1:** Baseline data

	Hypoxaemia (n = 229)		Normoxaemia (n = 269)		Hyperoxaemia (n = 491)		Severe hyperoxaemia (n = 200)		*P*-value
*Demographics*									
Age [years]	58	[43; 74]	61	[48; 75]	58	[41; 74]	51.5	[31; 69]	< 0.001
Age groups									
16–45	63	[27.5]	62	[23.0]	153	[31.2]	84	[42.0]	
> 45–65	82	[35.8]	95	[35.3]	150	[30.5]	57	[28.5]	
> 65–75	36	[15.7]	50	[18.6]	83	[16.9]	27	[13.5]	
> 75	48	[21.0]	62	[23.0]	105	[21.4]	32	[16.0]	0.008
Gender									
Female	50	[21.8]	61	[22.7]	146	[29.7]	74	[37.0]	
Male	179	[78.2]	208	[77.3]	345	[70.3]	126	[63.0]	0.001
*Vitals*									
SBP [mmHg]	129	[101; 155]	132	[112; 153]	131	[108; 151]	121	[103; 145]	0.011
SBP < 90mmHg	29	[12.8]	21	[7.8]	58	[11.8]	27	[13.6]	0.180
Pulse [bpm]	87	[72; 107]	88	[73; 101]	86	[72; 102]	88	[72; 101]	0.726
Pulse < 60/min	12	[5.3]	13	[4.9]	43	[8.8]	14	[7.1]	0.146
Pulse > 100/min	74	[32.6]	69	[25.7]	133	[27.2]	51	[25.8]	0.300
GCS	14	[10; 15]	14	[10; 15]	13	[3; 15]	5.5	[3; 14]	< 0.001
GCS < 9	44	[21.3]	56	[23.4]	159	[36.2]	95	[55.9]	< 0.001
RR [/min]	18	[15; 24]	18	[16; 23]	17	[15; 22]	16	[14; 23]	0.002
Bradypnea < 10/min	1	[0.6]	0	[0.0]	6	[1.7]	3	[2.2]	0.176
Tachypnea > 30/min	14	[8.3]	11	[5.5]	19	[5.3]	12	[8.9]	0.339
Temperature[°C]	37	[36; 37]	36	[36; 37]	36	[36; 37]	36	[36; 37]	0.579
SpO2 [%]	96	[92; 99]	97	[95; 100]	98	[96; 100]	100	[97; 100]	< 0.001
Intubation (prehospital)	41	[17.9]	56	[20.8]	151	[30.8]	100	[50.0]	< 0.001
*Injury characteristics*									
ISS	25	[19; 33]	26	[20; 34]	25	[21; 33]	25	[20.5; 34]	0.754
AIS head	2	[0; 4]	3	[0; 4]	3	[0; 4]	3	[0; 5]	0.019
AIS face	0	[0; 1]	0	[0; 1]	0	[0; 1]	0	[0; 2]	0.230
AIS neck	0	[0; 0]	0	[0; 0]	0	[0; 0]	0	[0; 0]	0.889
AIS thorax	3	[0; 4]	3	[0; 3]	2	[0; 3]	2	[0; 3]	< 0.001
AIS abdomen	0	[0; 0]	0	[0; 2]	0	[0; 1]	0	[0; 1]	0.977
AIS spine	2	[0; 2]	0	[0; 2]	0	[0; 2]	0	[0; 2]	0.248
AIS UE	0	[0; 2]	0	[0; 2]	0	[0; 2]	0	[0; 2]	0.880
AIS LE	0	[0; 3]	0	[0; 3]	0	[0; 3]	0	[0; 3]	0.780
AIS external	0	[0; 0]	0	[0; 0]	0	[0; 0]	0	[0; 0]	0.987

AIS, Abbreviated Injury Scale; bpm, beats per minute; GCS, Glascow Coma Scale; ISS, Injury Severity Score; LE, Lower Extremity; RR, Respiratory Rate; SpO2, oxygen saturation; SBP, Systolic Blood Pressure; UE, upper Extremity

**Table 2:** Outcome data and complications

	Hypoxaemia (n = 229)	Normoxaemia (n = 269)	Hyperoxaemia (n = 491)	Severe hyperoxaemia (n = 200)	P-value
**Complications**					
Any complications	90 [39.3]	92[34.2]	175 [35.6]	78 [39.0]	0.556
None	140 [61.1]	177 [65.8]	316 [64.4]	122 [61.0]	0.602
Stroke	1 [0.4]	1 [0.4]	0[0.0]	1 [0.5]	0.534
Myocardial infarction	3 [1.3]	2 [0.7]	1 [0.2]	0 [0.0]	0.160
Pulmonary embolism	6 [2.6]	8 [3.0]	12 [2.4]	4 [2.0]	0.926
Deep vein thrombosis	2 [0.9]	1 [0.4]	1 [0.2]	1 [0.5]	0.633
Pressure ulcer	2 [0.9]	1 [0.4]	2 [0.4]	0 [0.0]	0.576
Renal insufficiency	8 [3.5]	7 [2.6]	11 [2.2]	2 [1.0]	0.394
Abdominal distension	0 [0.0]	0 [0.0]	0 [0.0]	1 [0.5]	0.176
Wound infection	1 [0.4]	1 [0.4]	4 [0.8]	4 [2.0]	0.223
Pneumonia	31 [13.5]	23 [8.6]	48 [9.8]	22 [11.0]	0.298
Urinary tract infection	6 [2.6]	2 [0.7]	5 [1.0]	1 [0.5]	0.143
Sepsis	7 [3.1]	3 [1.1]	13 [2.6]	2 [1.0]	0.243
Compartment (extremity)	1 [0.4]	0 [0.0]	0 [0.0]	2 [1.0]	0.083
Compartment (abdomen)	0 [0.0]	0 [0.0]			
ALI/ARDS	0[0.0]	1 [0.4]	1 [0.2]	1 [0.5]	0.737
Cardiac arrest	1[0.4]	3 [1.1]	2 [0.4] 1	[0.5]	0.644
Multiorgan failure	0[0.0]	3 [1.1]	1 [0.2]	1 [0.5]	0.199
**Outcome**					
LOS [days]	8[5; 14]	10[7; 17]	11 [6; 18]	12.5 [7; 18.5]	0.001
LOS-ICU [days]	2.0[1.0; 5.1]	2.0[1.0; 5.0]	2.8 [1.0; 6.8]	3.8 [1.8; 9.0]	< 0.001
28-day mortality	36[15.7]	38[14.1]	68[13.8]	32 [16.0]	0.846

ALI/ARDS, Acute Lung Injury / Acute Respiratory Distress Syndrome; ICU, Intensive Care Unit; LOS, Length Of Stay

### Outcomes and complications

No significant difference was shown for 28-day mortality between the four groups with different oxygen partial pressure (*p* = 0.846, see Table 2).

Patients exhibiting severe hyperoxaemia experienced a significantly prolonged LOS with a median of 12.5 days (IQR 7–18.5) compared to normoxaemic patients, who had a median LOS of 10 days (7–17, *p* = 0.040). There was also a significant difference observed in LOS-ICU between the two groups, with 3.8 days (1.8–9) for patients with severe hyperoxaemia versus 2 days [1–5] for normoxaemic patients, yielding a *p*-value of < 0.001.

In total, 435 (36.6%) experienced complications during their hospitalization. Among all complications, pneumonia was the most common with 10.4%, followed by pulmonary embolism (2.5%) and renal insufficiency (2.4%) (Table 2). Overall, no significant differences were observed when comparing the four groups (Table 2).

In multivariable analysis, oxygen group was not associated with the primary outcome of 28-day mortality (Table 3) or LOS-ICU (supplemental Table 3). Severe hyperoxaemia patients had a statistical tendency towards a longer hospital stay (adjusted beta coefficient 2.23 [95% CI: − 0.32; 4.79], *p* = 0.087).

**Table 3:** Multivariable analysis for the primary outcome (28-day mortality)

28-day mortality	aOR	(95% CI)	p-value
Oxygen groups			
Hypoxaemia	1.47	[0.83; 2.60]	0.190
Normoxaemia [reference]	1.00		
Hyperoxaemia	0.92	[0.56; 1.52]	0.756
Severe hyperoxaemia	1.03	[0.56; 1.90]	0.917
Age [per year]	1.05	[1.04; 1.07]	< 0.001
SBP [per mmHg]*	0.99	[0.99; 1.00]	0.001
Intubation (prehospital)	4.21	[2.76; 6.43]	< 0.001
ISS [per point]	1.05	[1.03; 1.07]	< 0.001
AIS head	1.28	[1.14; 1.43]	< 0.001
AIS face	0.68	[0.54; 0.84]	< 0.001
AIS neck	1.30	[1.01; 1.66]	0.038

aOR, adjusted Odds Ratio; AIS, Abbreviated Injury Scale; ISS, Injury Severity Score; SBP, Systolic Blood Pressure

^*^0.6% of the values were imputed

### Non-linear association for PaO_2_ and 28-day mortality

**Fig. 2:**
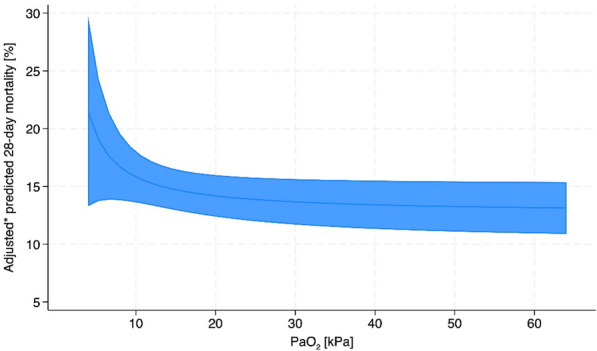
Visualization of the fractional polynomial regression with inverse-transformed PaO_2_ (= 1/PaO_2_); *p*-value = 0.060. Figure Description**:** The figure illustrates the relationship between arterial oxygen partial pressure (PaO₂) and adjusted predicted 28-day mortality. The shaded blue area represents the 95% confidence interval for the predicted mortality. At PaO₂ levels below 10 kPa, predicted 28-day mortality is high, indicating a greater risk of death. As PaO₂ increases, predicted mortality decreases rapidly, showing that even small improvements in oxygen levels can significantly reduce mortality risk. Beyond 20–30 kPa, further increases in PaO₂ result in only marginal decreases in mortality, suggesting diminishing returns at higher oxygen levels. *Abbreviations* aBGA, Arterial Blood Gas Analysis. *adjusted on age, ISS, AIS head, systolic blood pressure (with imputed values), and prehospital intubation

The inverse of PaO2 (1/PaO2) was the best predictor of 28-day mortality, based on varying PaO2 levels adjusted on age, ISS, AIS head, systolic blood pressure and prehospital intubation (*p* = 0.060, Fig. 2).

## Discussion

In this retrospective multicentre cohort study of 1,189 severely injured patients in Switzerland, both hypoxaemia and hyperoxaemia were frequently observed upon hospital admission. Hyperoxaemia was not associated with an increased 28-day mortality when compared to normoxaemia. However, the presence of severe hyperoxaemia showed a tendency with an extended hospital stay compared to normoxaemia patients.

While the detrimental effects of hypoxaemia are well recognized, the potential risks of hyperoxaemia have received less attention in previous research. Although prior studies have reported hyperoxaemia as a common occurrence in trauma patients [7], a finding in line with our data, its impact on patient outcomes remains insufficiently investigated.

This study revealed that patients with severe hyperoxaemia had prolonged stays both in hospital and in the ICU compared to normoxaemic patients. After adjusting for confounding variables such as the observable differences in baseline characteristics, the difference in LOS disappeared. However, we still observed a trend towards a longer hospital stay in severe hyperoxaemia patients. These findings are consistent with previous studies [15, 21]. For instance, Baekgaard et al. reported an increased mortality for patients with a FiO_2_ > 60% for more than 2 h within the first 24 h [21], while Douin et al. demonstrated an association between higher administered oxygen fractions during hyperoxaemia and mortality [15]. These results are particularly noteworthy considering that only short periods of hyperoxaemia within the first few hours after trauma may negatively affect outcomes [21]. However, all these studies, including ours, were retrospective analyses.

In contrast, a small randomized controlled trial involving 68 traumatic brain injury patients showed an improved neurological outcome when higher oxygen therapy was administered for the first 6 h after admission [14].

One limitation of this retrospective study is the uneven size of the groups and differences in baseline characteristics (age, gender, head trauma). Further, a notably higher number of hyperoxemic patients, including those with severe hyperoxemia, were intubated prehospital compared to normoxemic patients. It is known that intubated patients typically experience extended stays in the ICU and overall hospitalization duration. However, our study couldn’t discern whether the prolonged ICU and hospital stays resulted from prehospital intubation or were directly linked to hyperoxemia itself. Only patients with an aBGA were included which might have created a potential selection bias as patients without aBGA were notably less severe injured and had lower mortality and shorter hospital and ICU stay (Supplement Fig. 4).

A strength of this retrospective study is the multicentre design encompassing a large general trauma population in Switzerland with a complete dataset for primary outcome data.

This study, along with the diverging current literature highlights the urgent need for a large randomized controlled trial adequately powered to investigate the optimal oxygen administration and address confounders like prehospital intubation in trauma patients. The forthcoming results from the TRAUMOX2 (NCT05146700) and SAVE-O2 (NCT04534959) studies may provide valuable insights into this matter.

## Conclusion

This study indicate that hyperoxaemia is not associated with increased 28-day mortality compared to normoxaemia. Nonetheless, both moderate and severe hyperoxaemia are frequently detected in trauma patients, with severe hyperoxaemia exhibiting a tendency towards prolonged hospital stays compared to normoxaemia patients. Given the retrospective nature of our study, comprehensive randomized controlled trials are essential to fully assess the potential correlation between hyperoxaemia and outcomes in trauma patients.

### Supplementary Information

Additional file 1

## Data Availability

The datasets used and/or analysed during the current study are available from the corresponding author on reasonable request.

## Abbreviations

(a)BGA: (Arterial) blood gas analysis
AIS: Abbreviated injury scale
aOR: Adjusted odds ratio
ATLS: Advanced trauma life support
CO_2_: Carbon dioxide
FiO_2_: Inspired oxygen fraction
GCS: Glasgow coma scale
ICU: Intensive care unit
IQR: Interquartile range
ISS: Injury severity score
kPA: Kilopascal
KSSG: Hospital of Canton St. Gallen, Switzerland
LOS: Length of hospital stay
LOS ICU: Length of ICU stay
mmHg: Millimetre of mercury
PaCO_2_: Arterial partial pressure of carbon dioxide
PaO_2_: Arterial partial pressure of oxygen
PHTLS: Prehospital trauma life support
SaO_2_: Arterial oxygen saturation
SD: Standard deviation
SpO_2_: Oxygen saturation
WHO: World Health Organization
