# Supplementary material for: Hyperoxaemia in acute trauma is common and associated with a longer hospital stay: a multicentre retrospective cohort study
Source: Scand J Trauma Resusc Emerg Med. 2024 Aug 21;32:75. doi: 10.1186/s13049-024-01247-5 (PMC11340037; doi:10.1186/s13049-024-01247-5)
Supplement: Supplementary file 1 — Additional file 1 [file 13049_2024_1247_MOESM1_ESM.docx]

**Supplement**

#### Supplement table 1. Further injury characteristics according to oxygen group as numbers [%].

|  | **Hypoxaemia**  **(n=229)** | | | **Normoxaemia**  **(n=269)** | | | **Hyperoxaemia**  **(n=491)** | | | **Severe hyperoxaemia**  **(n=200)** | | | **P-value** | |
| --- | --- | --- | --- | --- | --- | --- | --- | --- | --- | --- | --- | --- | --- | --- |
| Severe (=AIS>2) head trauma | | 113 | [49.3] | 156 | | [58.0] | 280 | | [57.0] | 124 | | [62.0] | 0.057 | |
| Severe face trauma | | 11 | [4.8] | 11 | | [4.1] | 33 | | [6.7] | 9 | | [4.5] | 0.381 | |
| Severe neck trauma | | 4 | [1.7] | 9 | | [3.3] | 20 | | [4.1] | 5 | | [2.5] | 0.375 | |
| Severe thorax trauma | | 136 | [59.4] | 142 | | [52.8] | 228 | | [46.4] | 80 | | [40.0] | <0.001 | |
| Severe abdomen trauma | | 37 | [16.2] | 42 | | [15.6] | 75 | | [15.3] | 37 | | [18.5] | 0.765 | |
| Severe spine trauma | | 48 | [21.0] | 55 | | [20.4] | 94 | | [19.1] | 35 | | [17.5] | 0.798 | |
| Severe UE trauma | | 7 | [3.1] | 7 | | [2.6] | 14 | | [2.9] | 8 | | [4.0] | 0.834 | |
| Severe LE trauma | | 66 | [28.8] | 68 | | [25.3] | 136 | | [27.7] | 55 | | [27.5] | 0.834 | |
| AIS group (head) | |  |  |  | |  |  | |  |  | |  |  | |
| AIS 0-2 | | 116 | [50.7] | 113 | | [42.0] | 211 | | [43.0] | 76 | | [38.0] |  | |
| AIS 3 | | 30 | [13.1] | 36 | | [13.4] | 73 | | [14.9] | 28 | | [14.0] |  | |
| AIS 4-5 | | 83 | [36.2] | 120 | | [44.6] | 207 | | [42.2] | 96 | | [48.0] | 0.201 | |
| AIS group (face) | |  |  |  | |  |  | |  |  | |  |  | |
| AIS 0-2 | | 218 | [95.2] | 258 | | [95.9] | 458 | | [93.3] | 191 | | [95.5] |  | |
| AIS 3 | | 11 | [4.8] | 10 | | [3.7] | 32 | | [6.5] | 9 | | [4.5] |  | |
| AIS 4-5 | | 0 | [0.0] | 1 | | [0.4] | 1 | | [0.2] | 0 | | [0.0] | 0.595 | |
| AIS group (neck) | |  |  |  | |  |  | |  |  | |  |  | |
| AIS 0-2 | | 225 | [98.3] | 260 | | [96.7] | 471 | | [95.9] | 195 | | [97.5] |  | |
| AIS 3 | | 3 | [1.3] | 5 | | [1.9] | 13 | | [2.6] | 3 | | [1.5] |  | |
| AIS 4-5 | | 1 | [0.4] | 4 | | [1.5] | 7 | | [1.4] | 2 | | [1.0] | 0.740 | |
| AIS group (thorax) | |  |  |  | |  |  | |  |  | |  |  | |
| AIS 0-2 | | 93 | [40.6] | 127 | | [47.2] | 263 | | [53.6] | 120 | | [60.0] |  | |
| AIS 3 | | 73 | [31.9] | 102 | | [37.9] | 156 | | [31.8] | 52 | | [26.0] |  | |
| AIS 4-5 | | 63 | [27.5] | 40 | | [14.9] | 72 | | [14.7] | 28 | | [14.0] | <0.001 | |
| AIS group (abdomen) | |  |  |  | |  |  | |  |  | |  |  | |
| AIS 0-2 | | 192 | [83.8] | 227 | | [84.4] | 416 | | [84.7] | 163 | | [81.5] |  | |
| AIS 3 | | 17 | [7.4] | 20 | | [7.4] | 33 | | [6.7] | 14 | | [7.0] |  | |
| AIS 4-5 | | 20 | [8.7] | 22 | | [8.2] | 42 | | [8.6] | 23 | | [11.5] | 0.912 | |
| AIS group (spine) | |  |  |  | |  |  | |  |  | |  |  | |
| AIS 0-2 | | 181 | [79.0] | 214 | | [79.6] | 397 | | [80.9] | 165 | | [82.5] |  | |
| AIS 3 | | 27 | [11.8] | 34 | | [12.6] | 41 | | [8.4] | 20 | | [10.0] |  | |
| AIS 4-5 | | 21 | [9.2] | 21 | | [7.8] | 53 | | [10.8] | 15 | | [7.5] | 0.370 | |
| AIS group (UE) | |  |  |  | |  |  | |  |  | |  |  | |
| AIS 0-2 | | 222 | [96.9] | 262 | | [97.4] | 477 | | [97.1] | 192 | | [96.0] |  | |
| AIS 3 | | 7 | [3.1] | 7 | | [2.6] | 13 | | [2.6] | 8 | | [4.0] |  | |
| AIS 4-5 | | 0 | [0.0] | 0 | | [0.0] | 1 | | [0.2] | 0 | | [0.0] | 0.872 | |
| AIS group (LE) | |  |  |  | |  |  | |  |  | |  |  | |
| AIS 0-2 | | 163 | [71.2] | 201 | | [74.7] | 355 | | [72.3] | 145 | | [72.5] |  | |
| AIS 3 | | 31 | [13.5] | 31 | | [11.5] | 69 | | [14.1] | 31 | | [15.5] |  | |
| AIS 4-5 | | 35 | [15.3] | 37 | | [13.8] | 67 | | [13.6] | 24 | | [12.0] | 0.865 | |

**Abbreviations:** AIS, Abbreviated Injury Scale; LE, Lower Extremity; UE, Lower/Upper Extremity.

*Supplement table 2. Distribution of characteristics according to 28-day mortality*

|  | **N** | **Total**  **(n=1189)** | | **Alive after 28d**  **(n=1015)** | | **Dead within 28d (n=174)** | | **P-value** |
| --- | --- | --- | --- | --- | --- | --- | --- | --- |
| **DEMOGRAPHICS** |  |  |  |  |  |  |  |  |
| Age [years] | 1189 | 57 | [40; 73] | 56 | [37; 70] | 73 | [58; 82] | <0.001 |
| Age groups | 1189 |  |  |  |  |  |  |  |
| 16-45 |  | 362 | [30.4] | 339 | [33.4] | 23 | [13.2] |  |
| >45-65 |  | 384 | [32.3] | 345 | [34.0] | 39 | [22.4] |  |
| >65-75 |  | 196 | [16.5] | 153 | [15.1] | 43 | [24.7] |  |
| >75 |  | 247 | [20.8] | 178 | [17.5] | 69 | [39.7] | <0.001 |
| Gender | 1189 |  |  |  |  |  |  |  |
| Female |  | 331 | [27.8] | 274 | [27.0] | 57 | [32.8] |  |
| Male |  | 858 | [72.2] | 741 | [73.0] | 117 | [67.2] | 0.117 |
| **VITALS** |  |  |  |  |  |  |  |  |
| SBP [mmHg] | 1182 | 130 | [107; 151] | 130 | [109; 151] | 119.0 | [92; 149] | 0.001 |
| SBP <90mmHg | 1182 | 135 | [11.4] | 102 | [10.1] | 33 | [19.1] | 0.001 |
| Pulse [bpm] | 1182 | 87 | [72; 103] | 87 | [72; 102] | 89 | [71; 110] | 0.505 |
| Pulse <60/min | 1182 | 82 | [6.9] | 64 | [6.3] | 18 | [10.5] | 0.049 |
| Pulse >100/min | 1182 | 327 | [27.7] | 269 | [26.6] | 58 | [33.7] | 0.055 |
| GCS | 1055 | 13 | [3; 15] | 14 | [7; 15] | 3 | [3; 12] | <0.001 |
| GCS <9 | 1055 | 354 | [33.6] | 252 | [28.0] | 102 | [66.2] | <0.001 |
| RR [/min] | 861 | 18 | [15; 22] | 18 | [15; 23] | 15 | [14; 22] | 0.004 |
| Bradypnea <10/min | 861 | 10 | [1.2] | 8 | [1.1] | 2 | [1.8] | 0.509 |
| Tachypnea >30/min | 861 | 56 | [6.5] | 47 | [6.3] | 9 | [8.0] | 0.481 |
| Temperature[°C] | 937 | 36.4 | [36; 37] | 36.5 | [36; 37] | 36.2 | [36; 37] | 0.001 |
| SpO2 [%] | 1113 | 98 | [95; 100] | 98 | [95; 100] | 97.5 | [94; 100] | 0.012 |
| Intubation (prehospital) | 1189 | 348 | [29.3] | 244 | [24.0] | 104 | [59.8] | <0.001 |
| **INJURY CHARACTERISTICS** |  |  |  |  |  |  |  |  |
| ISS | 1189 | 25 | [20; 34] | 25 | [20; 33] | 29 | [25; 38] | <0.001 |
| AIS head | 1189 | 3 | [0; 4] | 3 | [0; 4] | 4 | [3; 5] | <0.001 |
| AIS face | 1189 | 0 | [0; 1] | 0 | [0; 1] | 0 | [0; 1] | 0.255 |
| AIS neck | 1189 | 0 | [0; 0] | 0 | [0; 0] | 0 | [0; 0] | 0.058 |
| AIS thorax | 1189 | 2 | [0; 3] | 2 | [0; 3] | 2.5 | [0; 3] | 0.362 |
| AIS abdomen | 1189 | 0 | [0; 1] | 0 | [0; 2] | 0 | [0; 0] | 0.066 |
| AIS spine | 1189 | 0 | [0; 2] | 0 | [0; 2] | 0 | [0; 2] | 0.405 |
| AIS upper extremity | 1189 | 0 | [0; 2] | 0 | [0; 2] | 0 | [0; 2] | 0.064 |
| AIS lower extremity | 1189 | 0 | [0; 3] | 0 | [0; 3] | 0 | [0; 2] | 0.026 |
| AIS external | 1189 | 0 | [0; 0] | 0 | [0; 0] | 0 | [0; 0] | 0.116 |
| **AIS GROUPS** |  |  |  |  |  |  |  |  |
| Severe head trauma | 1189 | 673 | [56.6] | 538 | [53.0] | 135 | [77.6] | <0.001 |
| Severe face trauma | 1189 | 64 | [5.4] | 56 | [5.5] | 8 | [4.6] | 0.619 |
| Severe neck trauma | 1189 | 38 | [3.2] | 29 | [2.9] | 9 | [5.2] | 0.109 |
| Severe thorax trauma | 1189 | 586 | [49.3] | 499 | [49.2] | 87 | [50.0] | 0.838 |
| Severe abdomen trauma | 1189 | 191 | [16.1] | 172 | [16.9] | 19 | [10.9] | 0.045 |
| Severe spine trauma | 1189 | 232 | [19.5] | 194 | [19.1] | 38 | [21.8] | 0.402 |
| Severe UE trauma | 1189 | 36 | [3.0] | 30 | [3.0] | 6 | [3.4] | 0.726 |
| Severe LE trauma | 1189 | 325 | [27.3] | 291 | [28.7] | 34 | [19.5] | 0.013 |
| Severe external trauma | 1189 | 0 | [0.0] | 0 | [0.0] | 0 | [0.0] | - |
| AIS group (head) | 1189 |  |  |  |  |  |  |  |
| AIS 0-2 |  | 516 | [43.4] | 477 | [47.0] | 39 | [22.4] |  |
| AIS 3 |  | 167 | [14.0] | 152 | [15.0] | 15 | [8.6] |  |
| AIS 4-5 |  | 506 | [42.6] | 386 | [38.0] | 120 | [69.0] | <0.001 |
| AIS group (face) | 1189 |  |  |  |  |  |  |  |
| AIS 0-2 |  | 1125 | [94.6] | 959 | [94.5] | 166 | [95.4] |  |
| AIS 3 |  | 62 | [5.2] | 55 | [5.4] | 7 | [4.0] |  |
| AIS 4-5 |  | 2 | [0.2] | 1 | [0.1] | 1 | [0.6] | 0.277 |
| AIS group (neck) | 1189 |  |  |  |  |  |  |  |
| AIS 0-2 |  | 1151 | [96.8] | 986 | [97.1] | 165 | [94.8] |  |
| AIS 3 |  | 24 | [2.0] | 17 | [1.7] | 7 | [4.0] |  |
| AIS 4-5 |  | 14 | [1.2] | 12 | [1.2] | 2 | [1.1] | 0.126 |
| AIS group (thorax) | 1189 |  |  |  |  |  |  |  |
| AIS 0-2 |  | 603 | [50.7] | 516 | [50.8] | 87 | [50.0] |  |
| AIS 3 |  | 383 | [32.2] | 334 | [32.9] | 49 | [28.2] |  |
| AIS 4-5 |  | 203 | [17.1] | 165 | [16.3] | 38 | [21.8] | 0.152 |
| AIS group (abdomen) | 1189 |  |  |  |  |  |  |  |
| AIS 0-2 |  | 998 | [83.9] | 843 | [83.1] | 155 | [89.1] |  |
| AIS 3 |  | 84 | [7.1] | 74 | [7.3] | 10 | [5.7] |  |
| AIS 4-5 |  | 107 | [9.0] | 98 | [9.7] | 9 | [5.2] | 0.108 |
| AIS group (spine) | 1189 |  |  |  |  |  |  |  |
| AIS 0-2 |  | 957 | [80.5] | 821 | [80.9] | 136 | [78.2] |  |
| AIS 3 |  | 122 | [10.3] | 102 | [10.0] | 20 | [11.5] |  |
| AIS 4-5 |  | 110 | [9.3] | 92 | [9.1] | 18 | [10.3] | 0.704 |
| AIS group (UE) | 1189 |  |  |  |  |  |  |  |
| AIS 0-2 |  | 1153 | [97.0] | 985 | [97.0] | 168 | [96.6] |  |
| AIS 3 |  | 35 | [2.9] | 30 | [3.0] | 5 | [2.9] |  |
| AIS 4-5 |  | 1 | [0.1] | 0 | [0.0] | 1 | [0.6] | 0.054 |
| AIS group (LE) | 1189 |  |  |  |  |  |  |  |
| AIS 0-2 |  | 864 | [72.7] | 724 | [71.3] | 140 | [80.5] |  |
| AIS 3 |  | 162 | [13.6] | 144 | [14.2] | 18 | [10.3] |  |
| AIS 4-5 |  | 163 | [13.7] | 147 | [14.5] | 16 | [9.2] | 0.042 |
| AIS group (external) | 1189 |  |  |  |  |  |  |  |
| AIS 0-2 |  | 1189 | [100.0] | 1015 | [100.0] | 174 | [100.0] | - |
| **COMPLICATIONS** |  |  |  |  |  |  |  |  |
| Any complications | 1189 | 435 | [36.6] | 358 | [35.3] | 77 | [44.3] | 0.023 |
| None | 1189 | 755 | [63.5] | 658 | [64.8] | 97 | [55.7] | 0.022 |
| Stroke | 1189 | 3 | [0.3] | 1 | [0.1] | 2 | [1.1] | 0.011 |
| Myocardial infarction | 1189 | 6 | [0.5] | 3 | [0.3] | 3 | [1.7] | 0.014 |
| Pulmonary embolism | 1189 | 30 | [2.5] | 27 | [2.7] | 3 | [1.7] | 0.467 |
| Deep vein thrombosis | 1189 | 5 | [0.4] | 3 | [0.3] | 2 | [1.1] | 0.108 |
| Pressure ulcer | 1189 | 5 | [0.4] | 4 | [0.4] | 1 | [0.6] | 0.734 |
| Renal insufficiency | 1189 | 28 | [2.4] | 17 | [1.7] | 11 | [6.3] | <0.001 |
| Abdominal distension | 1189 | 1 | [0.1] | 1 | [0.1] | 0 | [0.0] | 0.679 |
| Wound infection | 1189 | 10 | [0.8] | 10 | [1.0] | 0 | [0.0] | 0.189 |
| Pneumonia | 1189 | 124 | [10.4] | 103 | [10.1] | 21 | [12.1] | 0.444 |
| Urinary tract infection | 1189 | 14 | [1.2] | 14 | [1.4] | 0 | [0.0] | 0.119 |
| Sepsis | 1189 | 25 | [2.1] | 17 | [1.7] | 8 | [4.6] | 0.013 |
| Compartment (extremity) | 1189 | 3 | [0.3] | 1 | [0.1] | 2 | [1.1] | 0.011 |
| Compartment (abdomen) | 1189 | 0 | [0.0] | 0 | [0.0] | 0 | [0.0] | - |
| ALI/ARDS | 1189 | 3 | [0.3] | 3 | [0.3] | 0 | [0.0] | 0.473 |
| Cardiac arrest | 1189 | 7 | [0.6] | 3 | [0.3] | 4 | [2.3] | 0.001 |
| Multiorgan failure | 1189 | 5 | [0.4] | 1 | [0.1] | 4 | [2.3] | <0.001 |
| **OUTCOME** |  |  |  |  |  |  |  |  |
| LOS [days] | 1189 | 11 | [6; 17] | 12 | [7; 18] | 4 | [3; 8] | <0.001 |
| LOS-ICU stay [days] | 1143 | 2.5 | [1.0; 6.2] | 2.5 | [1.0; 6.8] | 2.2 | [1.0; 5.5] | 0.417 |
| 28-day mortality | 1189 | 174 | [14.6] | 0 | [0.0] | 174 | [100.0] | <0.001 |

**Abbreviations:** ALI/ARDS, Acute Lung Injury / Acute Respiratory Distress Syndrome; AIS, Abbreviated Injury Scale; bpm, beats per minute; GCS, Glascow Coma Scale; ICU, Intensive Care Unit; ISS, Injury Severity Score; LE, Lower Extremity; LOS, Length Of Stay; RR, Respiratory Rate; SpO2, oxygen saturation; SBP, Systolic Blood Pressure; Upper Extemity.

*Supplement table 3. Multivariable linear regression analysis for the secondary outcomes*

| **LOS [days]** | **Coef.** | **(95% CI)** | **p-value** |
| --- | --- | --- | --- |
| Oxygen groups |  |  |  |
| Hypoxaemia | -0.45 | [-2.89; 1.99] | 0.716 |
| Normoxaemia | 0.00 |  |  |
| Hyperoxaemia | 0.49 | [-1.57; 2.55] | 0.640 |
| Severe hyperoxaemia | 2.23 | [-0.32; 4.79] | 0.087 |
| Age [per year] | -0.07 | [-0.11; -0.03] | <0.001 |
| ISS | -0.11 | [-0.19; -0.02] | 0.011 |
| AIS UE | 1.32 | [0.50; 2.14] | 0.002 |
| AIS LE | 1.59 | [1.03; 2.16] | <0.001 |
| AIS head | 0.49 | [0.03; 0.95] | 0.036 |
| AIS face | 1.18 | [0.31; 2.06] | 0.008 |
| Temperature[per °C]* | -0.86 | [-1.59; -0.13] | 0.021 |
| Pulse [per bpm]* | 0.05 | [0.01; 0.08] | 0.010 |

| **LOS-ICU* [days]** | **Coef.** | **(95% CI)** | **p-value** |
| --- | --- | --- | --- |
| Oxygen groups |  |  |  |
| Hypoxaemia | 0.42 | [-0.65; 1.50] | 0.440 |
| Normoxaemia | 0.00 |  |  |
| Hyperoxaemia | 0.73 | [-0.19; 1.64] | 0.118 |
| Severe hyperoxaemia | 0.92 | [-0.24; 2.08] | 0.119 |
| Age [per year] | -0.03 | [-0.05; -0.01] | 0.002 |
| Gender | 1.40 | [0.61; 2.19] | 0.001 |
| Intubation (prehospital) | 1.85 | [0.98; 2.71] | <0.001 |
| Pulse [per bpm]* | 0.03 | [0.01; 0.04] | 0.002 |
| AIS head | 0.84 | [0.63; 1.06] | <0.001 |
| AIS face | 0.53 | [0.14; 0.91] | 0.008 |
| Temperature[per °C]* | -0.50 | [-0.83; -0.18] | 0.003 |
| AIS abdomen | 0.44 | [0.18; 0.70] | 0.001 |
| AIS spine | 0.39 | [0.15; 0.62] | 0.001 |
| AIS UE | 0.48 | [0.12; 0.84] | 0.009 |
| AIS LE | 0.38 | [0.15; 0.61] | 0.002 |

**Abbreviations:** AIS, Abbreviated Injury Scale; bpm, beats per minute; ICU, Intensive Care Unit; ISS, Injury Severity Index; LE, Lower Extremity; LOS, Length Of Stay; UE, Upper Extremity. * contained imputed value

*Supplement table 4. Distribution of characteristics of excluded patients without aBGA compared to included patients with aBGA*

|  |  | **Total** | | **aBGA** | | **no aBGA** | |  |
| --- | --- | --- | --- | --- | --- | --- | --- | --- |
|  | **N** | **(n=2301)** | | **(n=1198)** | | **(n=1103)** | | **P-value** |
|  |  |  |  |  |  |  |  |  |
| **DEMOGRAPHICS** |  |  |  |  |  |  |  |  |
| Age [years] | 2301 | 60 | [41; 75] | 57 | [40; 73] | 62 | [42; 77] | <0.001 |
| Age groups | 2301 |  |  |  |  |  |  |  |
| 16-45 |  | 669 | [29.1] | 364 | [30.4] | 305 | [27.7] |  |
| >45-65 |  | 703 | [30.6] | 387 | [32.3] | 316 | [28.6] |  |
| >65-75 |  | 373 | [16.2] | 199 | [16.6] | 174 | [15.8] |  |
| >75 |  | 556 | [24.2] | 248 | [20.7] | 308 | [27.9] | 0.001 |
| Gender | 2301 |  |  |  |  |  |  |  |
| Female |  | 683 | [29.7] | 333 | [27.8] | 350 | [31.7] |  |
| Male |  | 1618 | [70.3] | 865 | [72.2] | 753 | [68.3] | 0.039 |
|  |  |  |  |  |  |  |  |  |
| **VITALS** |  |  |  |  |  |  |  |  |
| SBP [mmHg] | 2291 | 134.0 | [114.0; 155.0] | 130.0 | [107.0; 151.0] | 138.0 | [120.0; 158.0] | <0.001 |
| SBP <90mmHg | 2291 | 175 | [7.6] | 136 | [11.4] | 39 | [3.5] | <0.001 |
| Pulse [bpm] | 2292 | 83 | [70; 98] | 87 | [72; 103] | 80 | [69; 93] | <0.001 |
| Pulse <60/min | 2292 | 182 | [7.9] | 82 | [6.9] | 100 | [9.1] | 0.052 |
| Pulse >100/min | 2292 | 478 | [20.9] | 328 | [27.5] | 150 | [13.6] | <0.001 |
| GCS | 2081 | 14 | [11; 15] | 13 | [3; 15] | 15 | [14; 15] | <0.001 |
| GCS <9 | 2081 | 430 | [20.7] | 355 | [33.4] | 75 | [7.4] | <0.001 |
| RR [/min] | 1789 | 18 | [15; 22] | 18 | [15; 22] | 18 | [15; 22] | 0.818 |
| Bradypnea <10/min | 1789 | 23 | [1.3] | 10 | [1.2] | 13 | [1.4] | 0.623 |
| Tachypnea >30/min | 1789 | 80 | [4.5] | 56 | [6.4] | 24 | [2.6] | <0.001 |
| Temperature[°C] | 1792 | 36.5 | [36.0; 37.0] | 36.4 | [35.9; 37.0] | 36.6 | [36.2; 37.0] | <0.001 |
| SpO2 [%] | 2152 | 98 | [95; 100] | 98 | [95; 100] | 98 | [95; 100] | 0.202 |
| Intubation (preclinical) | 2296 | 410 | [17.9] | 349 | [29.2] | 61 | [5.6] | <0.001 |
|  |  |  |  |  |  |  |  |  |
| **INJURY CHARACTERISTICS** |  |  |  |  |  |  |  |  |
| ISS | 2301 | 25 | [18; 29] | 25 | [20; 34] | 22 | [17; 25] | <0.001 |
| AIS head | 2301 | 3 | [0; 4] | 3 | [0; 4] | 3 | [0; 4] | 0.275 |
| AIS face | 2301 | 0 | [0; 1] | 0 | [0; 1] | 0 | [0; 1] | 0.199 |
| AIS neck | 2301 | 0 | [0; 0] | 0 | [0; 0] | 0 | [0; 0] | <0.001 |
| AIS thorax | 2301 | 1 | [0; 3] | 2 | [0; 3] | 0 | [0; 3] | <0.001 |
| AIS abdomen | 2301 | 0 | [0; 0] | 0 | [0; 1] | 0 | [0; 0] | <0.001 |
| AIS spine | 2301 | 0 | [0; 2] | 0 | [0; 2] | 0 | [0; 2] | <0.001 |
| AIS UE | 2301 | 0 | [0; 2] | 0 | [0; 2] | 0 | [0; 2] | 0.006 |
| AIS LE | 2301 | 0 | [0; 2] | 0 | [0; 3] | 0 | [0; 2] | <0.001 |
| AIS external | 2301 | 0 | [0; 0] | 0 | [0; 0] | 0 | [0; 0] | 0.142 |
|  |  |  |  |  |  |  |  |  |
| **AIS GROUPS** |  |  |  |  |  |  |  |  |
| Severe head trauma | 2301 | 1363 | [59.2] | 681 | [56.8] | 682 | [61.8] | 0.015 |
| Severe face trauma | 2301 | 121 | [5.3] | 66 | [5.5] | 55 | [5.0] | 0.575 |
| Severe neck trauma | 2301 | 50 | [2.2] | 38 | [3.2] | 12 | [1.1] | 0.001 |
| Severe thorax trauma | 2301 | 977 | [42.5] | 588 | [49.1] | 389 | [35.3] | <0.001 |
| Severe abdomen trauma | 2301 | 269 | [11.7] | 191 | [15.9] | 78 | [7.1] | <0.001 |
| Severe spine trauma | 2301 | 345 | [15.0] | 232 | [19.4] | 113 | [10.2] | <0.001 |
| Severe UE trauma | 2301 | 54 | [2.3] | 36 | [3.0] | 18 | [1.6] | 0.030 |
| Severe LE trauma | 2301 | 530 | [23.0] | 327 | [27.3] | 203 | [18.4] | <0.001 |
| AIS group (head) | 2301 |  |  |  |  |  |  |  |
| AIS 0-2 |  | 938 | [40.8] | 517 | [43.2] | 421 | [38.2] |  |
| AIS 3 |  | 372 | [16.2] | 168 | [14.0] | 204 | [18.5] |  |
| AIS 4-5 |  | 991 | [43.1] | 513 | [42.8] | 478 | [43.3] | 0.005 |
| AIS group (face) | 2301 |  |  |  |  |  |  |  |
| AIS 0-2 |  | 2180 | [94.7] | 1132 | [94.5] | 1048 | [95.0] |  |
| AIS 3 |  | 118 | [5.1] | 64 | [5.3] | 54 | [4.9] |  |
| AIS 4-5 |  | 3 | [0.1] | 2 | [0.2] | 1 | [0.1] | 0.780 |
| AIS group (neck) | 2301 |  |  |  |  |  |  |  |
| AIS 0-2 |  | 2251 | [97.8] | 1160 | [96.8] | 1091 | [98.9] |  |
| AIS 3 |  | 30 | [1.3] | 24 | [2.0] | 6 | [0.5] |  |
| AIS 4-5 |  | 20 | [0.9] | 14 | [1.2] | 6 | [0.5] | 0.002 |
| AIS group (thorax) | 2301 |  |  |  |  |  |  |  |
| AIS 0-2 |  | 1324 | [57.5] | 610 | [50.9] | 714 | [64.7] |  |
| AIS 3 |  | 673 | [29.2] | 385 | [32.1] | 288 | [26.1] |  |
| AIS 4-5 |  | 304 | [13.2] | 203 | [16.9] | 101 | [9.2] | <0.001 |
| AIS group (abdomen) | 2301 |  |  |  |  |  |  |  |
| AIS 0-2 |  | 2032 | [88.3] | 1007 | [84.1] | 1025 | [92.9] |  |
| AIS 3 |  | 125 | [5.4] | 84 | [7.0] | 41 | [3.7] |  |
| AIS 4-5 |  | 144 | [6.3] | 107 | [8.9] | 37 | [3.4] | <0.001 |
| AIS group (spine) | 2301 |  |  |  |  |  |  |  |
| AIS 0-2 |  | 1956 | [85.0] | 966 | [80.6] | 990 | [89.8] |  |
| AIS 3 |  | 184 | [8.0] | 122 | [10.2] | 62 | [5.6] |  |
| AIS 4-5 |  | 161 | [7.0] | 110 | [9.2] | 51 | [4.6] | <0.001 |
| AIS group (UE) | 2301 |  |  |  |  |  |  |  |
| AIS 0-2 |  | 2247 | [97.7] | 1162 | [97.0] | 1085 | [98.4] |  |
| AIS 3 |  | 51 | [2.2] | 35 | [2.9] | 16 | [1.5] |  |
| AIS 4-5 |  | 3 | [0.1] | 1 | [0.1] | 2 | [0.2] | 0.046 |
| AIS group (LE) | 2301 |  |  |  |  |  |  |  |
| AIS 0-2 |  | 1771 | [77.0] | 871 | [72.7] | 900 | [81.6] |  |
| AIS 3 |  | 260 | [11.3] | 164 | [13.7] | 96 | [8.7] |  |
| AIS 4-5 |  | 270 | [11.7] | 163 | [13.6] | 107 | [9.7] | <0.001 |
|  |  |  |  |  |  |  |  |  |
| **COMPLICATIONS** |  |  |  |  |  |  |  |  |
| Any complications | 2301 | 656 | [28.5] | 440 | [36.7] | 216 | [19.6] | <0.001 |
| None | 2301 | 1647 | [71.6] | 759 | [63.4] | 888 | [80.5] | <0.001 |
| Stroke | 2301 | 5 | [0.2] | 3 | [0.3] | 2 | [0.2] | 0.722 |
| Myocardial infarction | 2301 | 7 | [0.3] | 6 | [0.5] | 1 | [0.1] | 0.074 |
| Pulmonary embolism | 2301 | 39 | [1.7] | 30 | [2.5] | 9 | [0.8] | 0.002 |
| Deep vein thrombosis | 2301 | 6 | [0.3] | 5 | [0.4] | 1 | [0.1] | 0.125 |
| Pressure ulcer | 2301 | 6 | [0.3] | 5 | [0.4] | 1 | [0.1] | 0.125 |
| Renal insufficiency | 2301 | 45 | [2.0] | 29 | [2.4] | 16 | [1.5] | 0.093 |
| Abdominal distension | 2301 | 1 | [0.0] | 1 | [0.1] | 0 | [0.0] | 0.337 |
| Wound infection | 2301 | 13 | [0.6] | 10 | [0.8] | 3 | [0.3] | 0.072 |
| Pneumonia | 2301 | 163 | [7.1] | 125 | [10.4] | 38 | [3.4] | <0.001 |
| Urinary tract infection | 2301 | 26 | [1.1] | 14 | [1.2] | 12 | [1.1] | 0.855 |
| Sepsis | 2301 | 36 | [1.6] | 26 | [2.2] | 10 | [0.9] | 0.015 |
| Compartment (extremity) | 2301 | 5 | [0.2] | 3 | [0.3] | 2 | [0.2] | 0.722 |
| Compartment (abdomen) | 2301 | 0 | [0.0] | 0 | [0.0] | 0 | [0.0] | - |
| ALI/ARDS | 2301 | 3 | [0.1] | 3 | [0.3] | 0 | [0.0] | 0.096 |
| Cardiac arrest | 2301 | 9 | [0.4] | 7 | [0.6] | 2 | [0.2] | 0.122 |
| Multiorgan failure | 2301 | 7 | [0.3] | 5 | [0.4] | 2 | [0.2] | 0.304 |
|  |  |  |  |  |  |  |  |  |
| **OUTCOME** |  |  |  |  |  |  |  |  |
| LOS [days] | 2301 | 9 | [5; 15] | 11 | [6; 18] | 8 | [5; 12] | <0.001 |
| LOS-ICU [days] | 2046 | 1.6 | [0.8; 4.0] | 2.5 | [1.0; 6.3] | 1.0 | [0.5; 2.0] | <0.001 |
| 28-day mortality | 2300 | 266 | [11.6] | 175 | [14.6] | 91 | [8.3] | <0.001 |

**Abbreviations:** aBGA, arterial blood gas analysis; ALI/ARDS, Acute Lung Injury / Acute Respiratory Distress Syndrome; AIS, Abbreviated Injury Scale; bpm, beats per minute; GCS, Glascow Coma Scale; ICU, Intensive Care Unit; ISS, Injury Severity Score; LE, Lower Extremity; LOS, Length Of Stay; RR, Respiratory Rate; SpO2, oxygen saturation; SBP, Systolic Blood Pressure; Upper Extemity.
